# VASA expression suggests shared germ line dynamics in bivalve molluscs

**DOI:** 10.1007/s00418-017-1560-x

**Published:** 2017-04-06

**Authors:** Liliana Milani, Andrea Pecci, Fabrizio Ghiselli, Marco Passamonti, Simone Bettini, Valeria Franceschini, Maria Gabriella Maurizii

**Affiliations:** Department of Scienze Biologiche, Geologiche ed Ambientali, University of Bologna, Via Selmi 3, 40126 Bologna, Italy

**Keywords:** Multipotent cells, Gonads, Immunohistochemistry, *Anadara kagoshimensis*, *Mya arenaria*, *Crassostrea gigas*

## Abstract

Germ line segregation can occur during embryogenesis or after embryogenesis completion, with multipotent cells able to give rise to both germ and somatic cells in the developing juvenile or even in adulthood. These undifferentiated cells, in some animals, are self-renewing stem cells. In all these cell lineages, the same set of genes, among which *vasa*, appears to be expressed. We traced VASA expression during the peculiar gonad rebuilding of bivalves to verify its presence from undifferentiated germ cells to mature gametes in an animal taxon in which the mechanism of germ line establishment is still under investigation. We utilized antibodies produced against VASPH, VASA homolog of *Ruditapes philippinarum* (Subclass Heterodonta), to compare the known expression pattern of *R. philippinarum* to two species of the Subclass Pteriomorphia, *Anadara kagoshimensis* and *Crassostrea gigas*, and another species of the Subclass Heterodonta, *Mya arenaria*. The immunohistological data obtained support a conserved mechanism of proliferation of “primordial stem cells” among the simple columnar epithelium of the gut, as well as in the connective tissue, contributing to the seasonal gonad reconstitution. Given the taxonomic separation of the analyzed species, we suggest that the process could be shared in bivalve molluscs. The presence of germ cell precursors in the gut epithelium appears to be a feature in common with model organisms, such as mouse, fruit fly, and human. Thus, the comparative study of germ line establishment can add details on bivalve development, but can also help to clarify the role that VASA plays during germ cell specification.

**Electronic supplementary material:**

The online version of this article (doi:10.1007/s00418-017-1560-x) contains supplementary material, which is available to authorized users.

## Introduction

The germ line is a cell lineage that segregates early in the embryo of most animal species. Once an individual achieves sexual maturity, the germ cells inside the gonad begin to differentiate into specialized cells, the gametes, which in turn undergo fertilization. The genetic information stored in germ cells is the only one passed from parents to offspring, as the function of all other cells, namely the somatic cells, is to express their genetic content for the construction of the body architecture, without contributing directly to the transmission of DNA across generations (Gilbert 2010).

Due to its function, the germ line presents peculiar features, starting from the first germ cells to originate during development—the primordial germ cells (PGCs). These cells mainly (1) require a period of inactivity for their specification, then (2) become motile cells, and, at the end of this phase, (3) take up residence into the gonad.

1. In many studied species, when the early germinal/somatic fate determination occurs, a general repression of transcription is observed within the presumptive PGCs (Cinalli et al. 2008). A widespread explanation to this is that, while somatic cells organize and differentiate by actively transcribing and translating soma-specific factors, germ cells preserve their potency, before the onset of a germinal specific program (Nieuwkoop and Sutasurya 1981; Leatherman and Jongens 2003).
2. Once specified, PGCs are often located far from the gonad they will be part of, so they migrate across the embryo. This process has many shared features among taxa, and one of the most striking is the guidance property of some evolutionarily conserved molecules which act as chemoattractants or chemorepellents (Richardson and Lehmann 2010). PGC movement is supported by cellular extrusions that mediate the sensing of external environment and support the migration over the fibers of the extracellular matrix (Jaglarz and Howard 1995; Blaser et al. 2006).
3. Finally, PGCs reach an ultimate location where they combine with somatic cells that previously contributed to their chemoattraction, and together form the gonad. So far, it is commonly assumed that PGCs, encased in a somatic microenvironment called niche, become the germ line stem cells (GSCs). The continuous reciprocal signaling between somatic and germ cells determines the balance of symmetric division, which maintains the niche populated, and the asymmetric division, which pushes germ cells toward differentiation and meiosis (Morrison and Spradling 2008).

## The specification of germ cells

The processes that realize such developmental dynamics are at least two. In the majority of the species, factors for PGC specification are induced by proper concentration of extracellular signals synthesized by neighboring somatic cells. This mode is called epigenesis and is considered the basal specification mode (Extavour and Akam 2003). In some other species, instead, the oocytes store a collection of RNAs, proteins, mitochondria, and even mitochondrial components (Amikura et al. 2001; Isaeva and Reunov 2001), a mode of specification referred to as preformation, in which the set of determinants is generally called germ plasm. Even if with differences in timing and mode of action, preformation and epigenesis often share signal molecules; among them, the gene *vasa—*firstly discovered in *Drosophila* (Schüpbach and Wieschaus 1986a)—encodes the RNA helicase VASA.

Data from lophotrochozoans and deuterostomes support in some cases a germ line segregation after embryogenesis completion, with multipotent cells that give rise to both germ and somatic cells in the developing juvenile (Juliano et al. 2010). These undifferentiated cells remain in some cases mitotically quiescent until later in development, and, in some animals, they are self-renewing stem cells (Juliano et al. 2010). Also, among lophotrochozoans, planarian germ cells are continually segregated from population of self-renewing pluripotent adult stem cells called neoblasts (Newmark et al. 2008). As far as these studies are proceeding, the same set of genes, such as *vasa, nanos*, and *piwi* (Juliano et al. 2010), appears to specify and maintain PGCs as well as long-term multipotent progenitor cells. Recently, the term PriSCs, for primordial stem cells, was proposed (Solana 2013) to account for the extensive similarities observed among stem cells and germ cells in many animals. Accordingly, changes in the extent of self-renewal, proliferation, and developmental potential of PriSCs may produce the differences observed across animals (Solana 2013).

The study of germ line specification in the phylum Mollusca has been quite overlooked for years. The present knowledge, coming from studies on single species, indicates that molluscs exhibit the highly conserved cleavage mechanism of spiralian, in which most of the mesoderm derives from the 4d blastomere, including the germinal tissue (Conklin 1897). Moreover, since the majority of studied protostomes featured epigenesis (Extavour and Akam 2003), molluscs were also considered to share such mechanism, with some cases of preformation, considered as exceptions (Verdonk 1973; Dohmen and Lok 1975). More recently, new studies contributed to the discussion on the topic, bringing out an interesting comparison between gastropods, which tend to retain epigenesis, and bivalves, which show preformation as derived character (Obata and Komaru 2012). In parallel with this, genes and molecules involved in germ line specification—in particular *vasa* homologs*—*have been identified in several species at transcript (Fabioux et al. 2004; Swartz et al. 2008; Kranz et al. 2010) or protein level (Milani et al. 2011).

Since an overlapping set of genes acts in both multipotent precursors and germ line, the discrimination between these cell lineages can be difficult. We decided to trace VASA expression because it is required for both germ line development (Drosophila; Schüpbach and Wieschaus 1986b) and gametogenesis (Drosophila, *C. elegans*, and mouse; Kuznicki et al. 2000; Styhler et al. 1998; Tanaka et al. 2000). VASA is an evolutionarily conserved protein (Gustafson and Wessel 2010) whose activity probably involves ATP-dependent unwinding of mRNA duplexes that allows selective translation of mRNAs co-located with VASA (Lorsch 2002). In this way, only cells expressing VASA would be able to translate specific messengers that would ultimately affect cell fate. The pathway downstream VASA is poorly known and probably very branched, meaning that it involves the interaction of multiple signaling cascades. VASA enzymatic activity suggests a mechanism of action as a permissive switch necessary for transcription of subsequent factors, still involved in PGC fate determination, like, for example, *nanos* in *Drosophila* (Gavis et al. 1996).

Given its wide spectrum of expression during germ line development, VASA is a good marker to trace germ cells, from undifferentiated precursors to mature gametes. In this work, we utilized antisera produced against the VASA homolog of the Manila Clam *Ruditapes philippinarum* (Adams & Reeve, 1850) to explore VASA expression in the germ cells of three species sampled in June 2015: *Mya arenaria* (Linnaeus, 1758), *Crassostrea gigas* (Thunberg, 1793) and *Anadara kagoshimensis* (Tokunaga, 1906), and compared them to what was already observed for VASA expression in *R. philippinarum* germ line (Milani et al. 2015a, b). In all these species, germ line specification and/or gonad development has been studied by means of morphological approaches (Coe 1943; Stickney 1963; Devauchelle 1990). However, molecular targeting was performed at the transcript level on *C. gigas* (Fabioux et al. 2004), where PGCs, originating from the 4d mesentoblast of the early blastula, were identified, whereas in *R. philippinarum*, germ cells were identified and studied at the protein level (Milani et al. 2011, 2015a, b). This analysis is also useful to clarify the seasonal gonad rebuilding described in some bivalves (e.g., Galtsoff 1964; Devauchelle 1990; Yurimoto et al. 2008; Knigge et al. 2014). In these animals, the gonad appears as a transient anatomical structure that experiences a period of development during the reproductive season, when the body mass can more than double, and periods in which it is disassembled, during the sexual rest. Consequently, the sex of the animal, that lacks any secondary sexual character, can be assessed only in the reproductive season, while it is not possible during the sexual rest, when there are no gametes (Gosling 2003; Helm and Bourne 2004). The gonadic tissue consists of a series of connected tubules organized in sack-like structures, generally called acini, in which germ cells differentiate centripetally from the external border (the wall) to the center (the lumen), where mature gametes accumulate in the spawning season. This process is deeply affected by environmental and trophic conditions (Gosling 2003).

## Materials and methods

### Sampling

Alive specimens (3 of *M. arenaria*, 3 of *C. gigas*, and 5 of *A. kagoshimensis*) collected from the Sacca di Goro (Adriatic Sea, Northern Italy) were promptly sacrificed in order to preserve tissue morphology. Individuals were collected in June 2015; according to the available literature, this period corresponds approximately to the phase of sexual maturity for the studied molluscs, although with some range-exceptions (see details in "Discussion" section).

The analyzed species belong to two distantly related clades: *A. kagoshimensis* (family Arcidae) [According to the present classification, all the past records of *Scapharca inaequivalvis* in the Mediterranean Sea have to be referred to as *A. kagoshimensis* (Despalatović et al. 2013)] and *C. gigas* (family Ostreidae) are within Pteriomorphia, while *M. arenaria* (family Myidae) and *R. philippinarum* (family Veneridae) are within Heterodonta. *M. arenaria* and *R. philippinarum* are gonochoric species (Coe and Turner 1938; Devauchelle 1990). In *C. gigas*, sex determination seems to be affected by environmental conditions, but, in general, the species is referred to be protandric, with some cases of simultaneous hermaphroditism (Coe 1943). No published data are available at present for *A. kagoshimensis*, that is suggested to be protandric hermaphrodite, as *C. gigas* (Turolla E. personal communication).

Tissues from adults of each species were used for immunohistochemistry and western blotting. Dissected portions of about 0.5–1 cm^3^ were collected in proximity of the gonad, which is located in different parts of the body depending on the species. In *M. arenaria*, the gonad is located at the center of the visceral mass, traversed by several loops and branches of the digestive tube (Vlès 1909), a situation similar to that of *R. philippinarum* (Devauchelle 1990). During the dissection, a similar position was identified for *A. kagoshimensis* as well. In *C. gigas*, the gonad is located anterodorsally, in correspondence of the umbo, surrounding the digestive gland and a portion of the gut (Environment Agency 2007). After observation of gametic smear under optical microscope, the animals resulted to be three females of *M. arenaria*, two females and one male of *C. gigas*, and two females and three males of *A. kagoshimensis*.

### Analyses

#### Alignment of bivalve VASA proteins

**Fig. 1:**
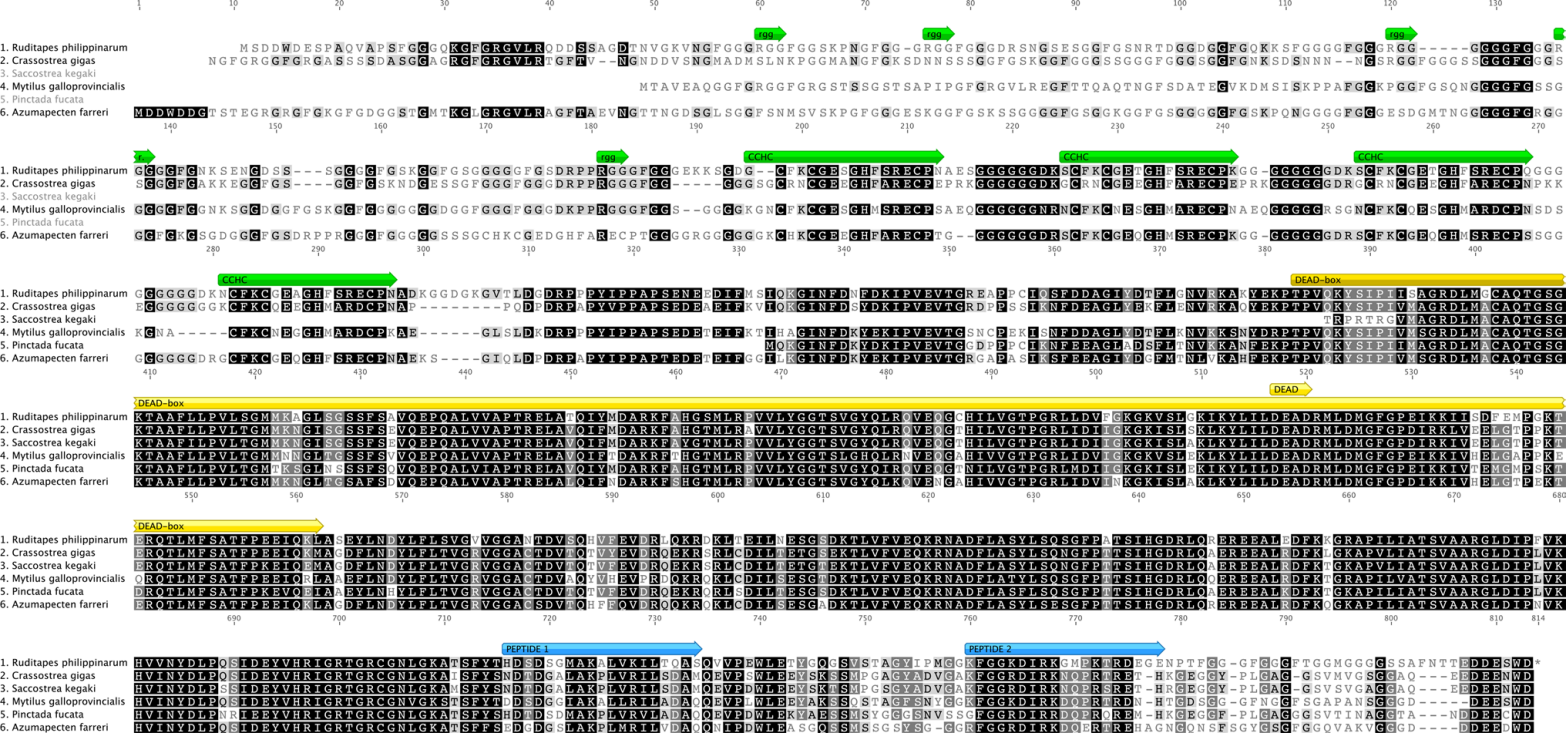
Alignment of bivalve VASA proteins. Name of the species present in GenBank with sequence accession number: *Ruditapes philippinarum* (JO110167.1), *Crassostrea gigas* (XP_011437246.1), *Saccostrea kegaki* (BAG55012.1), *Mytilus galloprovincialis* (BAJ15435.1), *Pinctada fucata* (BAM75192.1), *Azumapecten farreri* (ABE27759.1). VASA main domains are highlighted, as well as the peptides against which anti-VASPH antibodies were generated

We aligned bivalve VASA homologs having the following GenBank accession numbers: *R. philippinarum* (JO110167.1), *Crassostrea gigas* (XP_011437246.1), *Saccostrea kegaki* (BAG55012.1), *Mytilus galloprovincialis* (BAJ15435.1), *Perna canaliculus* (ACV04917.1), *Pinctada fucata* (BAM75192.1), *Azumapecten farreri* (ABE27759.1) using ClustalW (Larkin et al. 2007) (Fig. 1).

#### Primary antibody production

The antisera utilized in immunological analyses were produced against peptides of *R. philippinarum* VASA homolog (anti-VASPH). The chosen peptides resulted to be particularly conserved among VASA homologs of other bivalves present in GenBank (Fig. 1). These antibodies were named anti-HDS and anti-KFG from the first three amino acids of their respective immunogenic peptides [for details on antisera production see Milani et al. (2015a)].

#### Western blotting

Pools of male and female gonadic tissues from the same species were homogenized together (using an Ultra Turrax T25 Janke & Kunkel IKA- labortechnik) in a buffer containing 10 mM Tris–HCl, pH 7.5, 1 mM ethylene glycol-bis(2-aminoethyl ether)-N,N,N′,N′-tetraacetic acid (EGTA), 0.1% Sodium Dodecyl Sulfate (SDS). One protease inhibitor cocktail tablet (Complete Mini, Roche GmbH, Mannheim, GE) and 1 mM PMSF were added to 5 mL of the homogenization buffer before the use, to limit protein degradation. Total homogenates were centrifuged at 7500 rcf for 10 min at 4 °C in order to remove cellular debris. Proteins of gonadic extracts, contained in the supernatant, were aliquoted, quantified with Lowry method (Lowry et al. 1951) and stored at −80 °C. Gonadic extracts (20 and 30 μg) from each species were analyzed by sodium dodecyl sulfate polyacrylamide electrophoresis (SDS–PAGE, Laemmli 1970) using a 8.5% acrylamide gel. Some gel lanes were cut and processed for Coomassie Brilliant Blue staining, then they were digitalized by scanning.

For immunoblotting, proteins were transferred onto nitrocellulose membranes (Amersham Hybond Blotting Membranes, Buckinghamshire, UK). Non-specific binding sites were blocked with 5% dried skimmed milk (Bio-Rad Laboratories, Hercules, CA, USA), 3% Bovine Serum Albumin (BSA), in Tris-Buffered Saline (TBS) with 0.1% Tween-20 (Sigma) (TBS-Tw), 1 h 30 min at room temperature (RT), and subsequently washed for 30 min with TBS-Tw at RT. After blocking, membranes were incubated with polyclonal primary antisera against VASPH (anti-HDS or anti-KFG). The antisera were diluted 1:8000 for anti-HDS and 1:30,000 for anti-KFG in TBS-Tw and incubated over night at 4 °C, then for 1 h 30 min at RT. After rinsing for 30 min with TBS-Tw, we processed the membranes through incubation with goat anti-rabbit secondary antibody conjugated with horseradish peroxidase (Santa Cruz Biotechnology Inc. Santa Cruz, CA, USA) at the dilution of 1:5000 for 1 h at RT. The membranes were washed for 30 min, then they were detected using ECL Western Blotting Detection Reagents (Santa Cruz Biotechnology Inc. Santa Cruz, CA, USA), and exposed to Hyperfilm ECL (GE Healthcare Limited, Buckinghamshire, UK). Films were then digitalized by scanning. Negative controls were performed using the synthetic peptides used for the primary antibody production; each peptide was added to the antibody solution at a tenfold concentration before the incubation in order to bind every paratope of the primary antibody. In this way, the antibody is kept from binding its target, and the bands of interest are strongly attenuated.

#### Immunohistochemistry

Gonadic tissues were fixed in a solution containing 3.7% paraformaldehyde, 0.1% glutaraldehyde, 80 mM K-PIPES, 1 mM MgCl_2_, 5 mM EGTA, and 0.2% Triton X-100, pH 7, for 3 h 30 min. Then tissue was rinsed in phosphate buffered saline (PBS) (128 mM NaCl, 2 mM KCl, 8 mM Na_2_HPO_4_, 2 mM KH_2_PO_4_), pH 7.2, for 1 h with changes every 15 min. Afterward, samples were embedded in 7% agar. Sections of 100–150 μm thickness, obtained using a Lancer Vibratome Series 1000, were post-fixed with increasing concentrations of methanol (50–100%), rehydrated in Tris-Buffered Saline (TBS; 10 mM Tris–HCl, 155 mM NaCl), pH 7.4, and processed as free-floating sections. Unreacted aldehydes were reduced with 70 mM sodium borohydride in TBS, pH 7.4, for a 1 h 30 min at RT, followed by rinses for 1 h and 15 min in TBS. Antigenic sites were unmasked with 0.01% Pronase E (Merck Millipore) in PBS, pH 7.2, for 18 min at RT. Sections were rapidly washed with PBS, then samples were permeabilized adding TBS-1% Triton and left overnight at 4 °C. Non-specific protein-binding sites were blocked with 10% Normal Goat Serum (NGS) and 1% Bovine Serum Albumine (BSA) (Sigma) in TBS-0.1% Triton (TBS-0.1% T), pH 7.4, for 1 h 30 min. Then some sections were separately incubated with anti-HDS or anti-KFG, diluted 1:8000 and 1:30,000, respectively, with TBS-0.1% T containing 3% BSA, pH 7.4. The incubation lasted 72 h at 4 °C, followed by washes with TBS-0.1% T for 26 h. After washing, sections were incubated in the dark with the secondary antibody [1:400 polyclonal goat anti-rabbit Alexa Fluor 488 (Life Technologies, Carlsbad, CA, USA) in 1% NGS and 1% BSA in TBS-0.1% T, pH 7.4] for 32 h at 4 °C in the dark. After washing 24 h with several changes in TBS-0.1% T pH 7.4, a nuclear counterstaining was performed with 1 µM TO-PRO-3 nuclear dye (Life Technologies, Carlsbad, CA, USA) in PBS, pH 7.2, for about 10 min at RT in the dark, then the dye was washed in PBS and 30 min in TBS-0.1% T pH 7.4. All the immunostained sections were mounted in anti-fade medium [2.5% 1,4-diazabicyclo[2.2.2] octane (DABCO), 50 mM Tris, pH 8, and 90% glycerol]. Slides were stored horizontally at 4 °C in the dark. Images were recorded with a confocal laser scanning microscope (Leica confocal SP2 microscope), using Leica software. A total number of 161 sections was observed: 53 of *M. arenaria*, 49 of *C. gigas*, and 59 of *A. kagoshimensis*.

## Results

### Antibody specificity

The VASA alignment obtained using the amino acid sequences present in Genbank (Fig. 1) showed portions with a very low amino acid sequence divergence, ascribable to the most conserved known domains. Peptides chosen for antibody production resulted to be particularly conserved among VASA homologs of bivalves present in GenBank (accession numbers in Fig. 1).

**Fig. 2:**
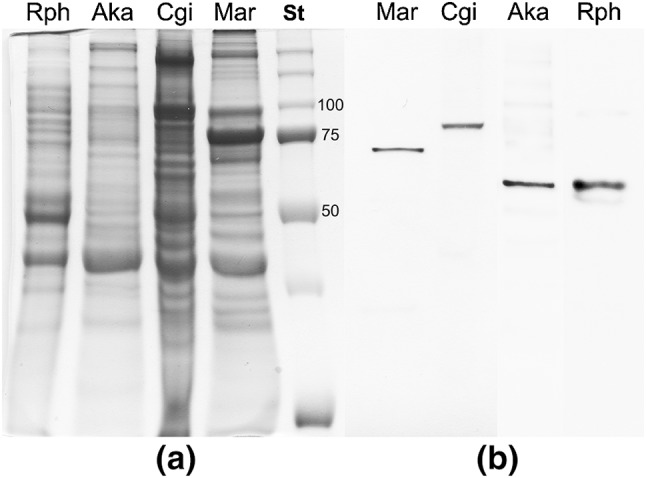
Anti-VASA specificity (**a**) Comassie Brilliant Blue gel staining. From *left* to *right: Ruditapes philippinarum* (Rph), *Anadara kagoshimensis* (Aka), *Crassostrea gigas* (Cgi), *Mya arenaria* (Mar), protein standard (St). (**b**) Western blots (anti-KFG). From *left* to *right*: Mar, Cgi, Aka, and Rph

Western blots using anti-VASPH gave specific results in *M. arenaria, C. gigas*, and *A. kagoshimensis* gonadic extracts (Fig. 2), and for each species a clear, single band was detected. According to the standards, the approximate molecular weight was 73 kDa in *M. arenaria*, 80 kDa in *C. gigas*, and 69 kDa in *A. kagoshimensis*. In the experimental controls, every major band was absent (Online Resource 1).

### Immunohistochemistry

**Fig. 3:**
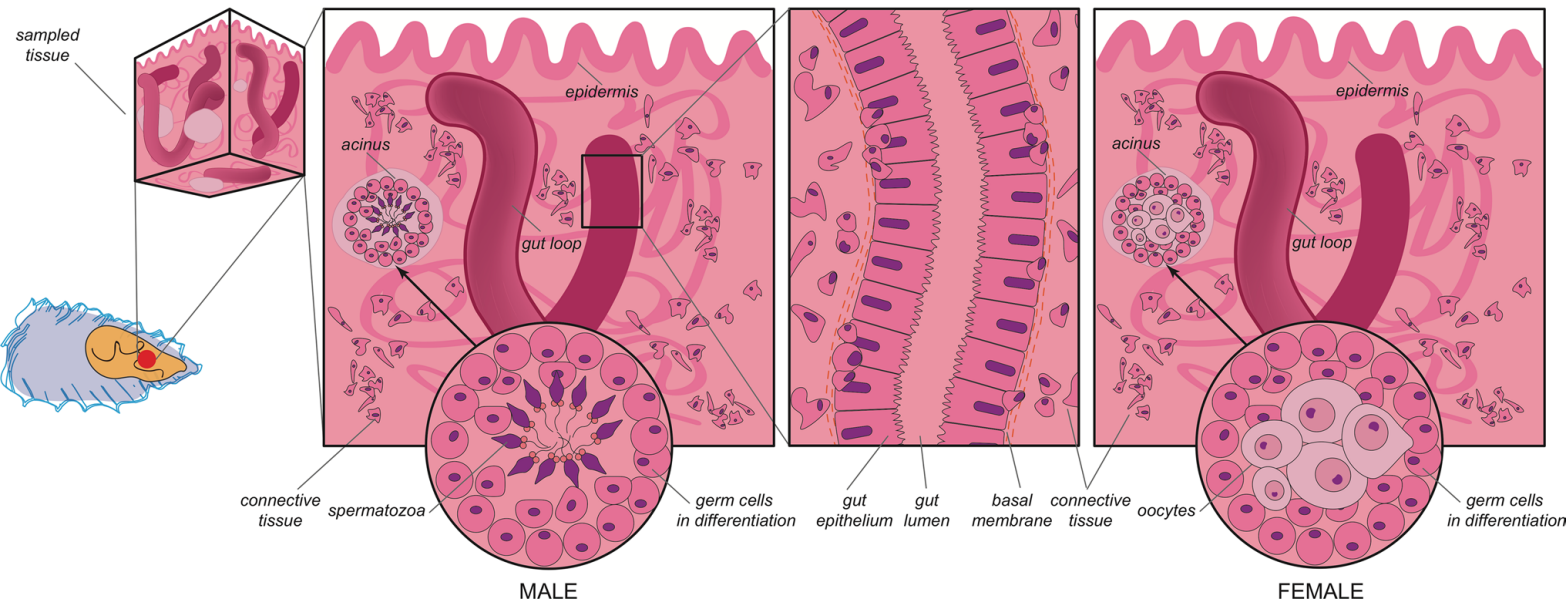
Schematic description of tissue organization in the analyzed bivalve species. The gonadic tissue in all the analyzed species is located close to the gut and consists of a series of connected tubules organized in acinus-like structures extended inside the connective tissue. The gut epithelium, consisting of a monolayer of batiprismatic cells, often shows ramifications with “loop” appearance, surrounded by connective tissue. In the basal side of the batiprismatic epithelium, near the basal membrane, roundish cells of small size are present. Similar cells are also dispersed among cells of connective tissue. In male and female acini, during the gonadic activity, germ cells differentiate centripetally from the wall to the lumen, where mature gametes are accumulated

A scheme of tissue organization in the analyzed bivalve species is illustrated in Fig. 3.

#### VASA immunodetection in *M. arenaria*

**Fig. 4:**
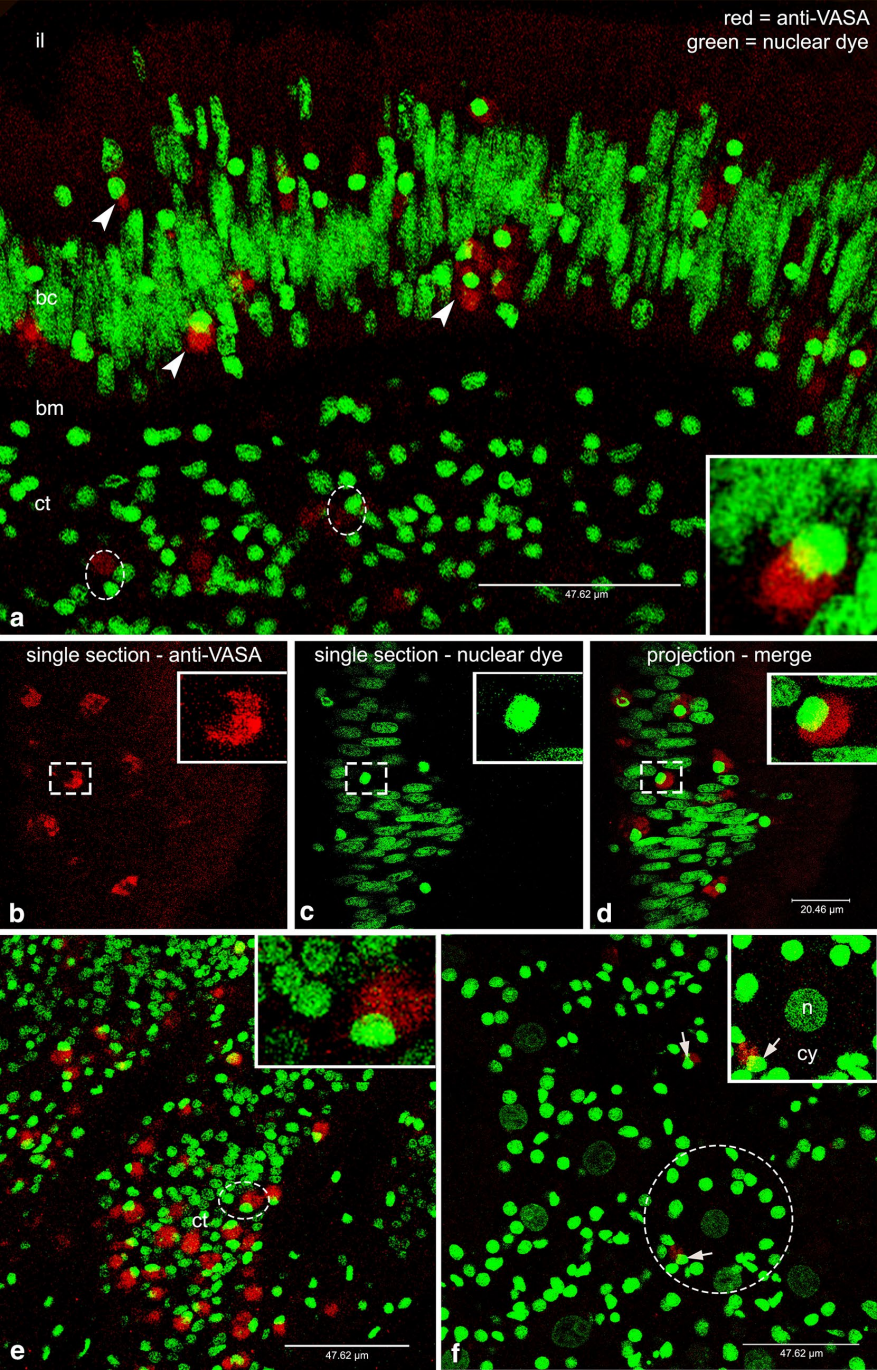
VASA-stained cells in *Mya arenaria*. **a** From the *top* to the *bottom*: intestinal lumen (il), batiprismatic cells (bc), basal membrane (bm), and connective tissue (ct). Several VASA-immunolabeled cells are evident within the intestinal wall (*arrowheads*) and in the connective tissue (*dashed* ovals). *Inset*: magnification of a stained cell inside the intestinal epithelium. **b** VASA staining (*red* channel) of a single optical section of the intestinal epithelium. *Inset*: magnification of the cell indicated by the *dashed rectangle* showing a strong VASA staining in the cytoplasm **c** Nuclear staining (*green* channel) of a single optical section of the intestinal epithelium. *Inset*: magnification of the cell indicated by the *dashed rectangle*. **d** Corresponding merge of the single section showed in **b** and **c. e** Many VASA-stained cells inside the connective tissue (ct). *Inset*: magnification of the VASA-stained cell in the dashed oval. **f** Gonadic tissue with many early developing oocytes. Few VASA-stained, early germ cells are visible around developing acini (*arrows*). Inset: magnification of the circled oocyte (n and cy: nucleus and cytoplasm of the oocyte) with a close immunolabeled cell (arrowhead). *Red* VASA staining, *green* nuclear staining

Sections of *M. arenaria* showed the gut surrounded by connective tissue. The epithelium consisted of a cellular wall separating a dark acellular zone—identified as the lumen—from the connective tissue (Fig. 4). The intestinal wall was recognizable by the monolayer in which it is organized, the stretched nuclear morphology and the presence of a dark continuous thin band between the epithelium and the connective tissue, namely the basal membrane (Fig. 4). Near this latter area of interface, round nuclei (Fig. 4a) were often present with an associated VASPH labeling (Fig. 4a). A magnification of this cell type showed the VASA labeling at one side of the cell, with respect to the nucleus (Fig. 4a–d). Groups of cells with a similar immunostaining were present inside the connective tissue (Fig. 4e and inset), while in other areas, female acini were visible (Fig. 4f). The acini were cut in cross and sagittal sections, supporting a branched tubular structure of the gonad. At a middle stage of maturity, medium yolked oocytes of 35–40 µm were often present inside the acini: oocytes had a tenuously stained chromatin, and a nucleolus (Fig. 4f).

#### VASA immunodetection in *C. gigas*

**Fig. 5:**
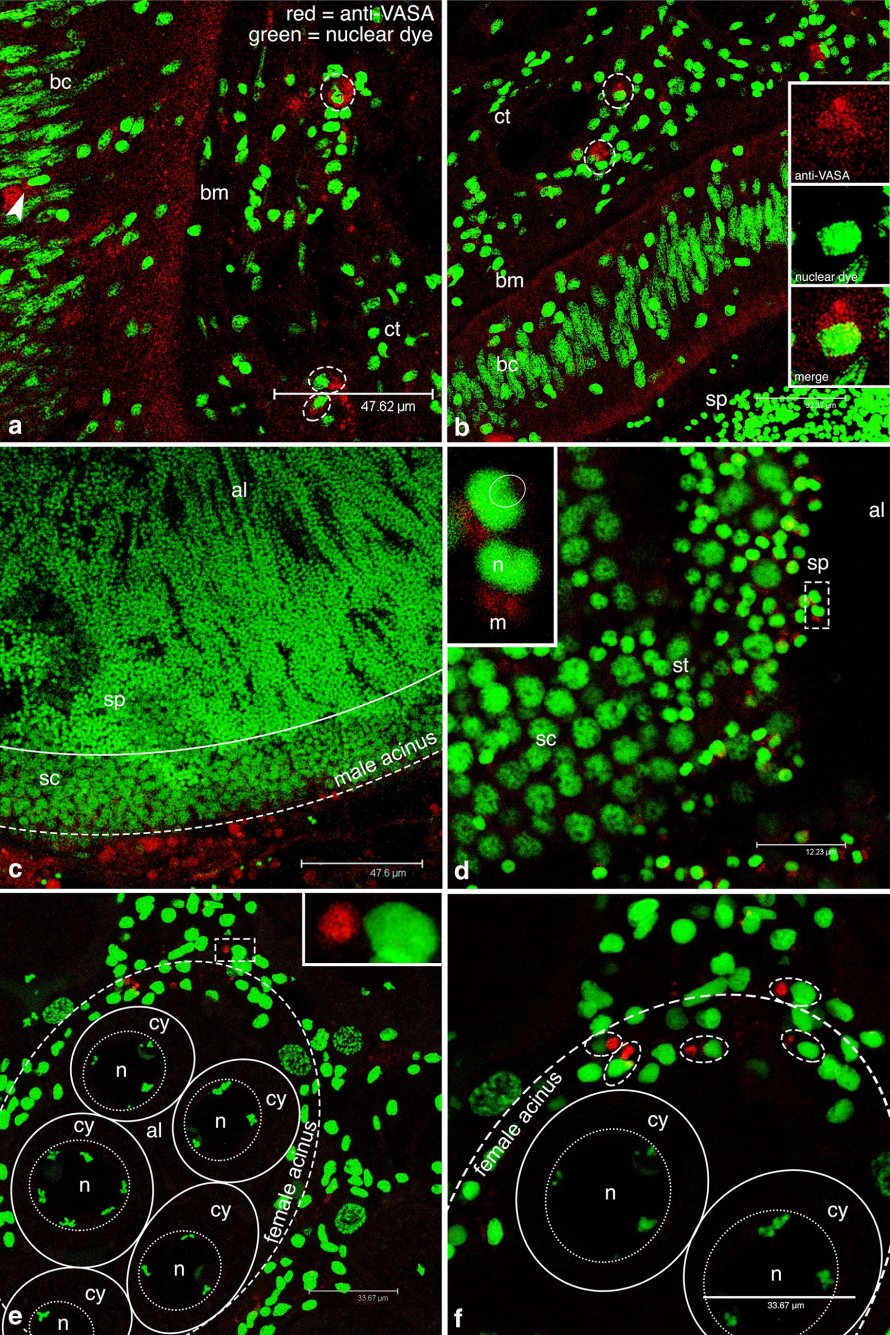
VASA-stained cells in *Crassostrea gigas*. **a** From the *left* to the *right*: intestinal epithelium with batiprismatic cells (bc), basal membrane (bm), and connective tissue (ct). In the intestinal wall, VASA-stained cells are present (*arrowhead*); in the connective tissue VASA-stained cells are circled. **b** A portion of an intestinal epithelium between the connective tissue (ct) and a male acinus full of spermatozoa (sp). Some VASA-stained cells (*circled*) in the connective tissue. *Inset*: a magnification of a circled VASA-stained cell. From the *top* to *bottom*: single optical section with *red* and *green* channels (anti-VASA and nuclear dye, respectively) and the corresponding merge. **c** Portion of male acinus. **d** Magnification of a detail of the acinus wall. From the periphery to the acinus lumen (al): spermatocytes (sc), spermatids (st), and spermatozoa (sp). Almost all the acinus volume is occupied by mature gametes (area surrounded by the *solid line*). *Inset*: magnification of the two spermatozoa (sp) in the dashed rectangle (a sperm head *fossa* is circled; sperm nucleus = n; midpiece mitochondria = m). **e** Female gonadic tissue with an acinus (*dashed circle*) full of oocytes (*solid circles*). Inside the acinus, oocytes show spiralized chromosomes in the nucleus (n) (cy = oocyte cytoplasm). Around the acinus, VASA-immunolabeled cells are present. Inset: magnification of the VASA-stained cell in the dashed rectangle. **f** A magnification of the acinus shown in (**e**) with some VASA-stained cells (dashed ovals) at the acinus periphery. *Red* VASA staining, *green* nuclear staining

In *C. gigas*, we found a weak anti-VASA signal in labeled cells within the gut epithelium and in the connective tissue (Fig. 5a, b). In male acini, several stages of spermatogenesis were recognizable from the nuclear morphology (Fig. 5c, d). The spermatozoa were recognizable from an intense bright green signal, revealing very condensed chromatin and a round-shaped nucleus. At one side of the nucleus, the VASA labeling was present in correspondence of the midpiece (Fig. 5d). In female acini, yolked oocytes with slightly irregular shape were present at various stages of differentiation, reaching up to 60 µm (Fig. 5e). The nuclear morphology of these oocytes often showed spiralized bichromatidic chromosomes, at the end of their first meiotic division. Round VASA-labeled cells—similar to those identified in *M. arenaria—*were present around the acini (Fig. 5e, f).

#### VASA immunodetection in *A. kagoshimensis*

**Fig. 6:**
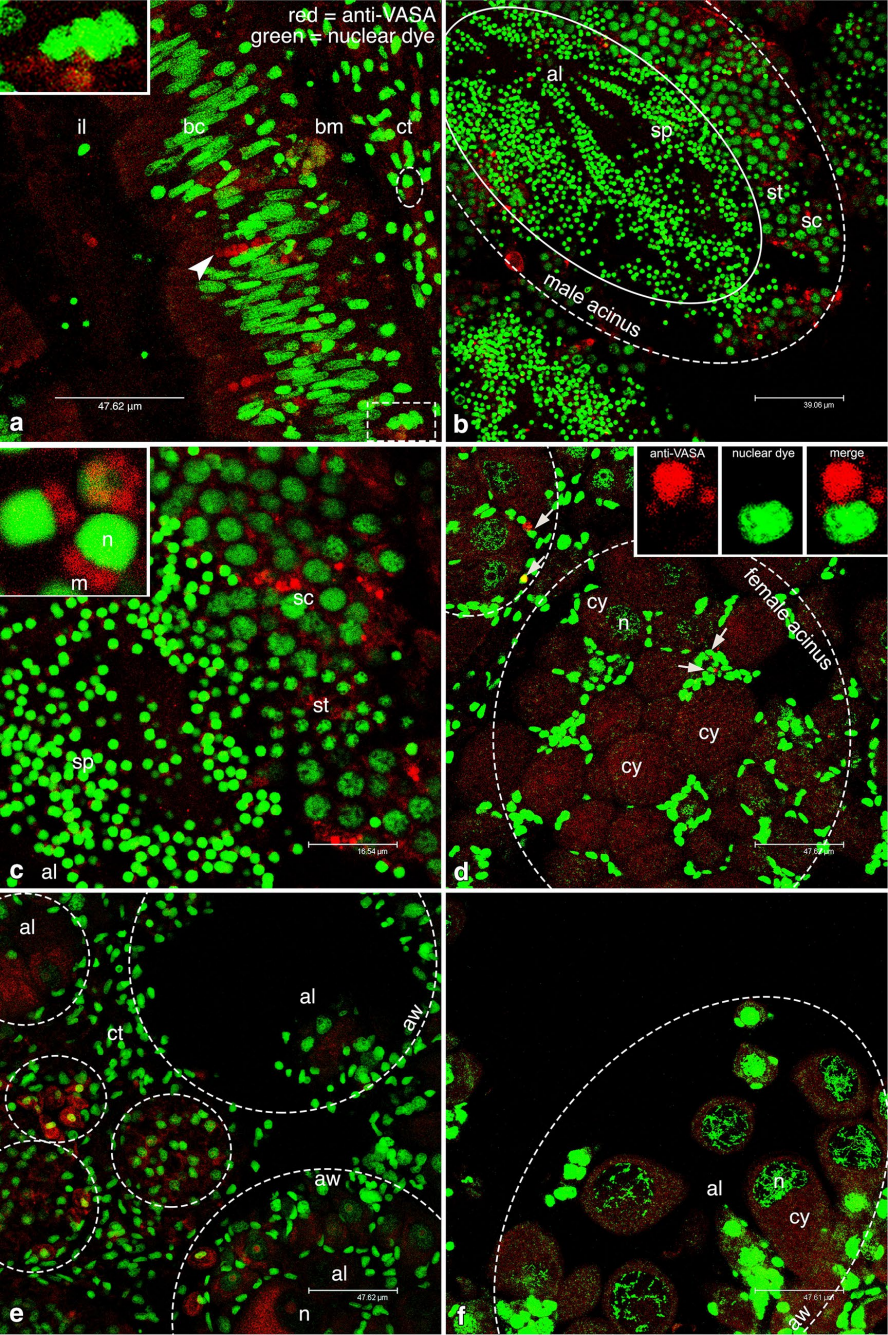
VASA-stained cells in *Anadara kagoshimensis*. **a** Intestinal wall with VASA-labeled cells (*arrowhead*). From the *left* to the *right*: intestinal lumen (il), batiprismatic cells (bc), basal membrane (bm), and connective tissue (ct). In the connective tissue VASA-immunolabeled cells are present (*dashed oval*). *Inset* magnification of the VASA-stained cells in the *dashed rectangle*. **b** Male acinus showing the centripetal organization of germ cells; the cells located at the periphery show a stronger VASA signal. From the periphery to the acinus lumen (al): spermatocytes (sc), spermatids (st), and spermatozoa (sp). The volume occupied by mature gametes is circled (*solid line*). **c** Magnification of a portion of the acinus shown in **b**. *Inset* magnified sperm heads (sperm nucleus = n) showing VASA-stained mitochondrial midpiece (m; five mitochondria can be counted). **d** Female acini (circled) containing several oocytes. VASA-labeled cells are present at acinus periphery (*arrows*). *Inset*: magnification of a VASA-stained cell (*arrow*): single optical section with *red* and *green* channels (anti-VASA and nuclear dye, respectively) and the corresponding merge. **e** Female gonadic tissue showing several acini (*circled*) of different dimension. Acinus wall (aw) and acinus lumen (al) are indicated. Early oocytes with a stronger cytoplasmic VASA staining are located close to the acinus wall. **f** Female acinus, sectioned at lumen level (al) showing many oocytes (n and cy: nucleus and cytoplasm of oocytes, respectively). *Red* VASA staining, *green* nuclear staining

*Anadara kagoshimensis* showed an intestine organized similarly to the previous species. At the basal pole of the monolayer, several cells, packed among the columnar epithelium, were immunostained (Fig. 6a and inset). This condition was present only in certain areas of the gut epithelium, while, in other areas, the intestine was devoid of labeled cells. In the connective tissue, few VASA-immunolabeled cells with a round nucleus were present.

Male acini were sectioned both longitudinally and transversally (Fig. 6b, c), and they showed a centripetal organization, with external labeled spermatocytes (Fig. 6b, c). During the differentiation process of germ cells, the VASA labeling became gradually accumulated at one side of the cell, and in spermatozoa—that were disposed in cordons (Fig. 6b)—it appeared condensed into the four–five rounded mitochondria of the midpiece (Fig. 6c, inset). Female acini contained oocytes showing a variably condensed nucleus (Fig. 6d–f)—sometimes with a slightly stained nucleolus (Fig. 6e)—and with a dimension ranging from 15 to 60 µm (Fig. 6d–f). Smaller oocytes had a clear VASA staining throughout the cytoplasm (Fig. 6e); VASA-labeled cells—similar to those identified in *M. arenaria—*were present around the acini.

Bigger oocytes instead showed a diffused faint staining (Fig. 6d, f), and sometimes an evident *pedunculus* connected them to the acinus wall (Fig. 6f). Cells with the VASA labeling concentrated at one side of the cytoplasm, near to a round nucleus, were visible close to the acinus wall (Fig. 6d, e).

Controls with only the secondary antibody gave no reaction positivity. In all the species, we never found the two gamete types in the same section, supporting the absence of simultaneous hermaphrodites.

## Discussion

### VASA is expressed in putative primordial stem cells

With the present work, we provide evidence that VASA-immunolabeled cells within the intestinal wall are commonly found across bivalve molluscs as well, corroborating what was previously observed in *R. philippinarum* (Milani et al. 2015a).

In the last years, the functional role of VASA has been related not only to germ cells, but also to other cell types (Gustafson and Wessel 2010; Lasko 2013; Yajima and Wessel 2015). For example, the snail *Ilyanassa obsoleta*, as well as other lophotrochozoans, show post-embryonic germ line segregation from multipotent progenitor cells (Juliano et al. 2010). In this snail, self-renewing stem cells originated from the 4d-cell lineage contribute to larval mesoderm and endoderm, but it is uncertain so far whether these cells remain into adulthood. However, evidence from another mollusc, *Sphaerium striatinum* (bivalvia, subclass Heterodonta), suggests that these cells retain their uncommitted morphology until adult gonad formation (Woods 1931). *vasa* mRNA is expressed in the 4d lineage (Swartz et al. 2008; Kranz et al. 2010), supporting the conclusion that these genes are expressed in multipotent cells of these molluscs (Juliano et al. 2010).

We here propose that VASA-labeled cells in the gut are PriSCs, primordial stem cells that can be precursors of both PGCs and cells of the somatic lineage (Solana 2013). PriSCs can be considered as a reservoir of stem cells expressing genes of the germline multipotency program (GMP) (e.g., *vasa, nanos, piwi*), program that would operate in both multipotent cells and germ cells (Juliano et al. 2010). These cells are capable of self-renewing, and can account for gonad rebuilding, that in these animals takes place every year at the beginning of the reproductive season, while, during the sexual rest, there are no morphological/anatomical differences between males and females. The re-formation of the gonad, that at maturity accounts for almost the whole body mass, implies an extremely high rate of cell proliferation, whose dynamics are only recently started to be matter of investigation. The fact that VASA-stained cells are present around gonadic acini, as well as in early developing germ cells, suggests that VASA is a germ line marker also in these bivalve species.

**Fig. 7:**
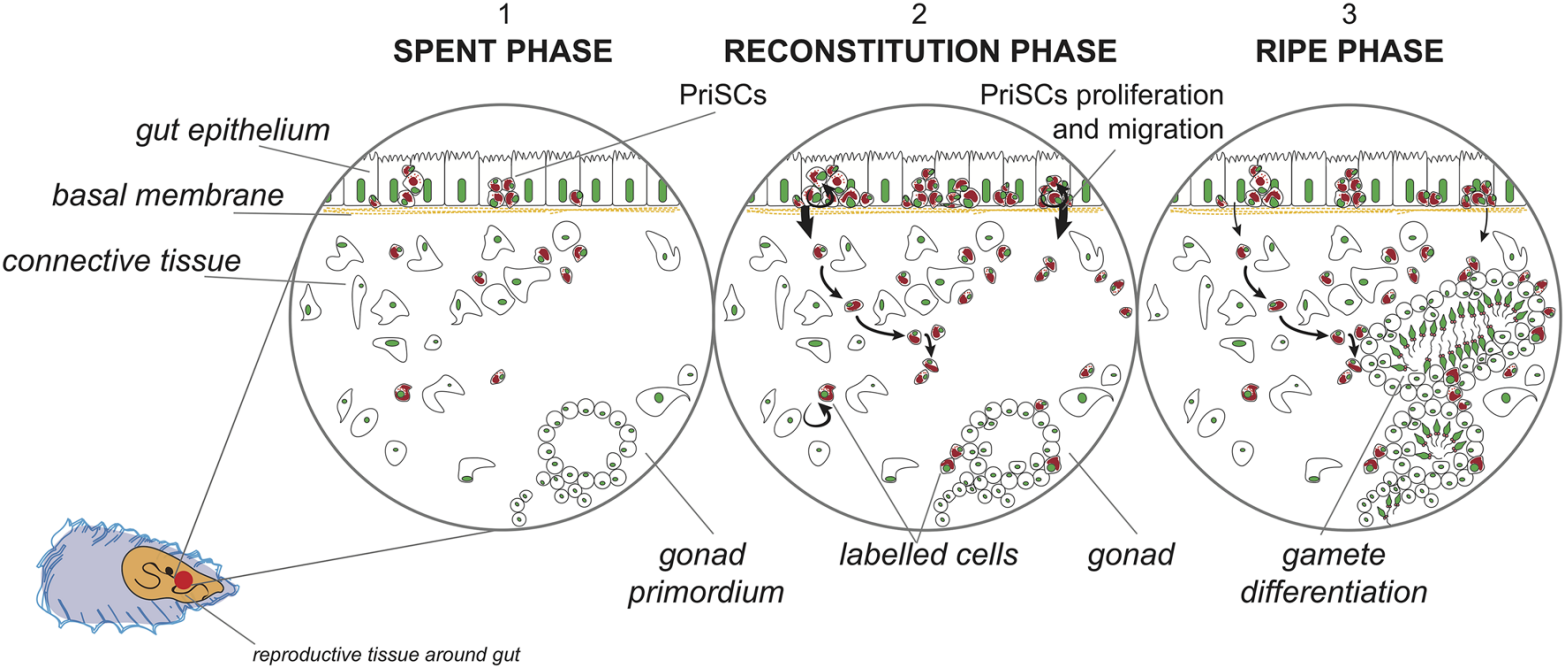
Visual abstract of our hypotheses on germ line dynamics during the annual reproductive cycle of bivalves. (1) Spent phase: few stained PGCs are localized in the intestine among batiprismatic cells, and other few stained germ cells can be found in the connective tissue; simple-structured acinus-like formation can be rarely found in the connective tissue. (2) Reconstitution phase: strongly immunostained PGCs massively proliferate among batiprismatic cells. Next, germ cells massively migrate to the connective tissue where they form new acini. (3) Ripe phase: mature acini are full of gametes. In males, the spermatozoon mitochondrial midpiece appears slightly VASA-stained. The VASA staining (in *red*) is usually accumulated at one side of the cell cytoplasm

The variable number of identifiable PriSCs observed in the analyzed species (see Figs. 4, 5, 6 for comparisons) can be related to the sexual maturity period being species-specific: different species of bivalves that are sampled simultaneously in the same environment can be at different stages of gonadal development. We suppose that the intestinal wall represents a niche from where PriSCs annually proliferate and migrate to the connective tissue, participating to gonad reconstitution (refer to Fig. 7 for a visual abstract of our hypothesis), but the timing of these activities—that occur before the emergence of developed and organized acini in the connective tissue—are not synchronous across the analyzed species. The intestine is an endo-mesodermal-derived organ, and its involvement in germ line dynamics has been reported for at least two model organisms: the mouse and the fruit fly. During mouse development, PGCs in the hindgut migrate through the epithelium to the mesoderm (Molyneaux et al. 2001). In *Drosophila*, PGCs early originated at the posterior pole of the embryo and passively carried onto the posterior midgut epithelium, pass through the intestinal wall to the mesoderm by diapedesis (Dansereau and Lasko 2008). A last yardstick that highlights the association between the intestine and germ line specification is given by the study of Mamsen et al. (2012), where human PGCs were immunochemically detected among the cells of the hindgut, tracing the germ cell markers OCT4 and c-Kit. The images of human embryos processed 4 and 5 weeks post-conception look very similar to those obtained in our study, suggesting that the developing intestine probably represents a conserved environment necessary for the onset and/or maintenance of germinal fate. Given the information present in bibliography, the intestinal epithelium represented the best candidate as a possible reservoir of germ cells, so we sampled tissue portions that included the gonad and the intestine, that are closely associated in the analyzed species.

Western blot gave only one specific band for each species, supporting the antibody specificity. A further clue about the identification of VASA in the immunodetected proteins is provided by the detection of the unique 80 kDa band in *C. gigas*. The molecular weight well matches the in silico translation of the mRNA sequence isolated by Fabioux et al. (2004) (79.960 kDa; http://www.uniprot.org/uniprot/Q6TEC0), study in which *vasa* transcript was put in relationship to germ line specification. While the DEAD-box domain and some portions of the C-terminus appear to be quite conserved among the analyzed VASA homologs (Fig. 1), the N-terminus is very variable both in length and amino acid composition, and this may be a reason for the different molecular weights (Fig. 2). We have to specify that, given the available data, it is not possible to have a reliable expected value because transcripts in database are often incomplete, and it is known that transcripts can experience post-transcriptional and post-translational modifications, so that the molecular weight resulting from in silico calculation of nucleotide sequences may not correspond to the true value.

Our interpretation of VASA-immunolabeled cells in the analyzed bivalves as cells being able to specify germ cells rests on the fact that (Milani et al. 2015b): (i) in these species, the gonad is rebuilt at every reproductive season and is disassembled at the end of it, and the proliferation of VASA-stained cells in the intestinal epithelium appears to be correlated to the stage of gonad maturation, and (ii) the antibody stained the same type of cells with the same localization in each analyzed species.

### VASA localization is in accordance with the phase of gonad maturation

As enlisted above, the proliferation of VASA-stained cells in the intestinal epithelium is correlated to the stage of gonad maturation. In all the four species, gonadic activity is seasonal and culminates when individuals are ready to spawn gametes. The reported spawning season according to the literature is: (i) June - August for *M. arenaria* (Cross et al. 2012: data referred to Southeast coast of Ireland, and confirmed for Northern Adriatic Sea, Turolla E. personal communication); (ii) March–April for *C. gigas* (Héral and Deslous-Paoli 1990: data referred to Northern Adriatic Sea, to be extended to June in the Goro lagoon, as supported by the present work); (iii) end of June–August for *A. kagoshimensis* (Campioni and Sbrenna 1993: data referred to Northern Adriatic Sea); (iv) June–September for *R. philippinarum* (Sbrenna and Campioni 1994: data referred to Northern Adriatic Sea). Periods of gonad rebuilding followed by further spawning events in the same year are documented of indicated by indirect observations (Arakawa 1990; Devauchelle 1990; Kang et al. 2003; Cross et al. 2012 and references therein).

The germ line stage observed in *M. arenaria* is in accordance with the specimens being in the period just after spawning (Fig. 4), before the following spawning event of the same year, so the expectation is to have many developing acini that can show an unclear morphology. Accordingly, no mature acini were found, but only early oocytes were present, since we observed unstained oocytes of 35–40 µm while, at maturity, oocytes are reported to be around 70 µm (Coe and Turner 1938). This condition is the most similar to *R. philippinarum* (Milani et al. 2015a), that spawns in the same period (Sbrenna and Campioni 1994). We detected a few proliferating PriSCs in the intestinal epithelium, while many of them were present in the connective tissue where new acini will form before the following spawning event. Early germ cells originated from PriSCs located in the connective tissue start proliferating and then undergo meiotic divisions. In this phase of *M. arenaria* life cycle, oocytes are starting the differentiation process, each one surrounded by few VASA-stained early germ cells (two in Fig. 4f). Those are probably the germ cells already activated and in proliferation. As proliferation and differentiation proceed, more complex and recognizable acini full of gametes will form. Accordingly, in the other two species, VASA-stained, early germ cells around acini appear to be more numerous (Figs. 6d, 5e, f).

Differently to what reported for *M. arenaria, A. kagoshimensis* germ line state indicated that the analyzed individuals were approaching a spawning event, showing acini with many differentiating germ cells and a large amount of mature gametes (Fig. 6). In *A. kagoshimensis*, we found oocytes of different dimensions: given the sampling period, it is likely that the observed 60 µm oocytes (Fig. 6f), showing a faint VASA staining, were at the end of vitellogenesis, while smaller oocytes (Fig. 6e), showing a stronger VASA staining, were probably still developing (Campioni and Sbrenna 1993). The observed difference in strength between the signal coming from oocytes with different size is probably due to VASA dilution in the larger and more developed ones. So, while *M. arenaria* (Fig. 4) is at the beginning of gamete production for its second spawning event, *A. kagoshimensis* is undergoing a fully active gamete production, thus, the signal for differentiation is at a high level, justifying an higher VASA content in oocytes (Fig. 6e). In male acini, inside the lumen, round-headed spermatozoa arranged in rows, as described by Yurimoto et al. (2008), presented four–five mitochondria as reported for the congeneric species *Anadara broughtoni* (Zhu et al. 2008).

*C. gigas*, that is characterized by an anticipated spawning season in comparison to the other analyzed species, appeared to be close to the final spawning event of the year. The oyster specimens showed an almost arrested proliferation, with very few undifferentiated germ cells in the connective tissue and a majority of mature gametes (Fig. 5). Female acini showed mature eggs of about 60 µm (Arakawa 1990; Leclerc et al. 2000 and references therein; Salinas-Flores et al. 2008), arrested in prophase I (according to the nuclear morphology and chromatin condensation; Fig. 5e). The male mature acini of *C. gigas* (Fig. 5c) present a large mass of spermatozoa, recognizable by their morphology (Gràcia Bozzo et al. 1993), and a relatively smaller mass of differentiating germ cells when compared to *A. kagoshimensis* (Fig. 6b), so that almost all the acinus volume is occupied by sperm. Around some large acini, it was possible to visualize stained cells (Fig. 5c) probably ascribable to PriSCs still present in the surrounding connective tissue. At this stage, in *C. gigas* there is no need to keep producing mature gametes, so the signal for differentiation, as indicated by VASA expression, is low or absent in spermatogonia, spermatocytes, and spermatids. Instead, *A. kagoshimensis* is still forming new gametes, as can be inferred by the reduced portion of the acinus occupied by sperm, and germ cell differentiation is still ongoing, as indicated by the high level of VASA expression.

For all the species analyzed here, the reproductive periods reported in literature match our observations. Only for *C. gigas* we recorded mature specimens beyond the known mature/spawning phase, indicating an extended spawning period, a possibility that is in accordance with the above mentioned natural fluctuation of seasonality.

### VASA is localized in the mitochondrial midpiece of the spermatozoon and in early oocytes

Interestingly, the spermatozoa analyzed in this study showed VASA expression in the midpiece, a localization also reported in the fish medaka (Yuan et al. 2014). At small magnification (e.g., Fig. 5c), it is difficult to visualize the staining that instead becomes evident at a higher magnification (e.g., Fig. 5d and inset). This is also due to the fact that part of the spermatozoa visualized in one section can have the mitochondrial midpiece placed in an adjacent section, at different levels on the z-axis. The labeling had the shape of defined round mitochondria, as observed in *R. philippinarum* (Milani et al. 2015a), and this can account for a tight association between mitochondria and VASA, similarly to what is known for mouse (Onohara et al. 2010). So midpiece mitochondria are detected and visible thanks to VASA immunolabeling (Figs. 5, 6).

The oocytes observed in this study were usually lacking VASA labeling (see for example, *M. arenaria* Fig. 4f, *C. gigas* Fig. 5e, f, *A. kagoshimensis* Fig. 6d, f). Only rarely (see *A. kagoshimensis* Fig. 6e) it was possible to see some cytoplasmic staining in oocytes. As previously observed, the different gonadal developmental stage is likely the cause of such differences. The three species were sampled on the same day, but being the reproductive cycles not synchronous, some variability in VASA expression is expected. Nonetheless, it would be extremely difficult to sample different species at the very same stage, also because, besides the rough datum about the period of spawning known for each species, the environmental component has to be considered too, and it may vary every year.

In our observations, we did not find evidence of a localized germ plasm in oocytes, however, because of the presence of VASA staining in the cytoplasm at one side of the nucleus in PriSCs and early germ cells, and the difficulties of germ plasm detection in mature oocytes by immunohistochemistry due to yolk storing that can prevent a successful labeling, we cannot exclude a preformation mode of germ line specification. Nonetheless, as deeply discussed in previous studies (e.g., Extavour 2007), preformation and epigenesis are not intended as mutually exclusive.

## Conclusions

The immunohistological data obtained support a similar mechanism of gonad reconstitution for the analyzed bivalves, starting from the seasonal proliferation of PriSCs among the simple columnar epithelium of the intestine, followed by their migration to the connective tissue in which they form acini full of gametes at different stages of differentiation (Fig. 7). The gonadic tissue reabsorption at the end of the reproductive season would end the process. According to what was observed in some bivalves during the resting period (spent phase), it is conceivable that some PriSCs (Solana 2013), as well as early germ cells, can remain in the connective tissue from the previous season, and start forming acini when the following reproductive season is approaching (Fig. 7). In Mytilus (Obata et al. 2010) and oysters (Sano et al. 2015), for example, *vasa* transcripts were detected with a low signal also during the spent season in the area in which the gonad forms.

We covered in previous works (Milani et al. 2011, 2015a, b) almost all the gonad rebuilding phases in *R. philippinarum* (the species used for comparison), so it is now possible to give a first description of similarities in different species. Given the taxonomic separation of the analyzed species, pertaining to two highly divergent clades, we suggest that the observed migration mechanism might be a shared feature of bivalve molluscs. In this context, it would be interesting to further investigate the presence and function of VASA isoforms in bivalves, as well as their supposed multifunctionality. Also, the study of bivalve reproductive biology, especially by mean of a comparative approach, can add details on their mechanism of development (Obata and Komaru 2012), but can also help to depict a more comprehensive overview of the role that regulators as VASA play.

## Electronic supplementary material

Below is the link to the electronic supplementary material. Online Resource 1 Anti-VASA specificity control.

Supplementary material 1 (TIF 5402 KB)
