# Supplementary figures and images for: VASA expression suggests shared germ line dynamics in bivalve molluscs
Source: Histochem Cell Biol. 2017 Apr 6;148(2):157–71. doi: 10.1007/s00418-017-1560-x (PMC5508042; doi:10.1007/s00418-017-1560-x)

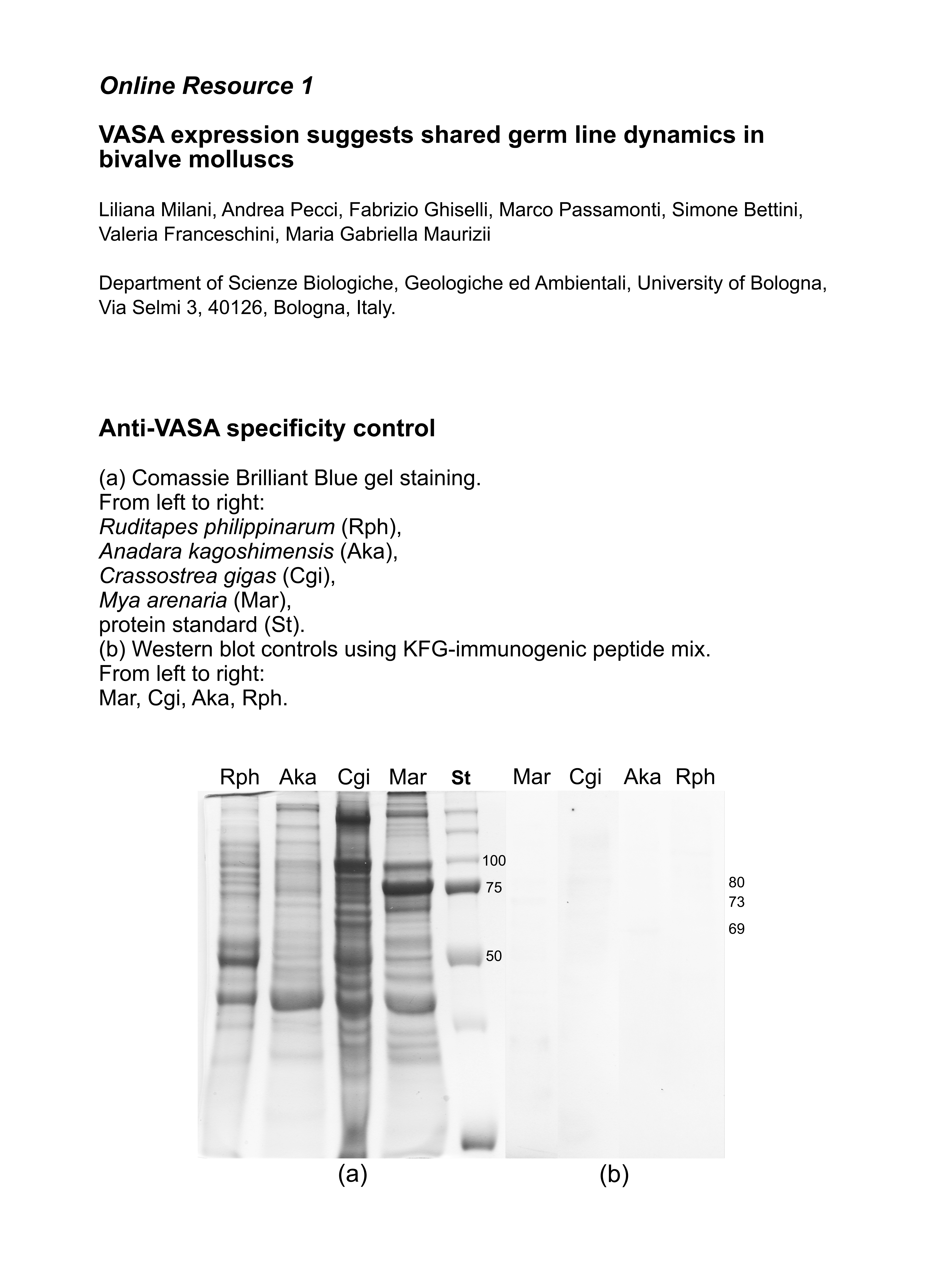

Supplement: Supplementary file 1 — Supplementary material 1 (TIF 5402 KB) [file 418_2017_1560_MOESM1_ESM.tif]
